# Time-based capnography detects ineffective triggering in mechanically ventilated children

**DOI:** 10.1186/s13054-019-2583-6

**Published:** 2019-09-04

**Authors:** Robert G. T. Blokpoel, Alette A. Koopman, Jefta van Dijk, Frans H. C. de Jongh, Johannes G. M. Burgerhof, Martin C. J. Kneyber

**Affiliations:** 1 Department of Paediatrics, Division of Paediatric Intensive Care, Beatrix Children’s Hospital, University Medical Center Groningen, University of Groningen, Internal Postal Code CA 62, P.O. Box 30.001, 9700 RB Groningen, the Netherlands; 2 Faculty of Science and Technology, University of Twente, Enschede, the Netherlands; 3 Department of Epidemiology, University Medical Center Groningen, University of Groningen, Groningen, the Netherlands; 4 Critical care, Anesthesiology, Peri-operative and Emergency medicine (CAPE), University of Groningen, Groningen, the Netherlands

To the Editor,

Ineffective triggering has been associated with an increased morbidity although a direct cause-effect relationship remains to be determined [1]. The ability of physicians to detect these events, merely using ventilator flow- and pressure-time scalars, was demonstrated to be quite low [2]. Several attempts have been made to automatically quantify patient-ventilator interaction, but most methods require monitoring additional signals, e.g. the electrical activity of the diaphragm or the oesophageal pressure [3, 4]. As time-based capnography is recommended for routine monitoring in ventilated patients and thus easily available, we sought to explore if ineffective patient inspiratory efforts could also be recognised in the time-based capnogram, providing the physician an additional tool for recognising ineffective triggering at the bedside.

For this purpose, we studied two cohorts. The first cohort was a retrospective analysis of previous collected data in which patient-ventilator interaction was quantified [5]. Patients in the first study cohort underwent a 5-min recording of the ventilator flow-time and pressure-time scalars, electrical activity of the diaphragm (dEMG) and time-based capnogram. In the second prospective cohort, patients underwent a 5-min recording of the ventilator flow-time, pressure-time, oesophageal pressure (Poes) and time-based capnogram. In both cohorts, patient ineffective trigger efforts (i.e. increase in dEMG or a negative deflection in the Poes without cycling the ventilator) were correlated with deflections in phase III or the β-angle of the time-based capnogram.

Fifty-five patients (34 boys, 21 girls) were analysed. Forty-one (75%) were admitted because of respiratory failure. Median age was 3.6 [1.6–16.0] months and median weight 6.0 [4.6–9.5] kg. Patients had been ventilated for a median of 3.8 [2.3–5.3] days before being studied. In 84% (46), patients were ventilated using a pressure A/C mode of ventilation. In the first cohort, 3823 breaths were analysed. One hundred and fifty-five of 213 trigger errors were recognisable in the flow- and pressure-time scalars, dEMG tracing and time-based capnogram (sensitivity 72.77%, specificity of 99.97%). There were no negative deflections recognised in the time-based capnogram in 50/58 (27%) events because the flow remained < 0 L/min. In the second cohort, 5365 breaths were analysed. Five hundred and thirty-seven of the 555 trigger errors were recognised in the time-based capnogram and the flow-, airway pressure- and oesophageal pressure-time scalars (sensitivity 96.76%, specificity 99.92%). In this cohort, there were no negative deflections visible in the time-based capnogram in 16/18 (3.24%) events because the flow remained < 0 L/min.

To our best knowledge, this is the first paediatric report that trigger errors can be detected in the time-based capnogram. When comparing deflections in the time-based capnogram against patient neural breathing drive (i.e. dEMG) and muscle effort (i.e. Poes), we found that if a patient was able to generate an inspiratory flow > 0 L/min that also became positive during the expiratory phase, deflections in the time-based capnogram identified ineffective triggering (Figs. 1 and 2). The caveat with this method is that trigger errors could not be picked up if the flow did not become positive. This may be overcome by taking the degree of negative deflections in the Poes measurements into account. Therefore, we think this is a promising approach that warrants further investigation.

**Fig. 1:**
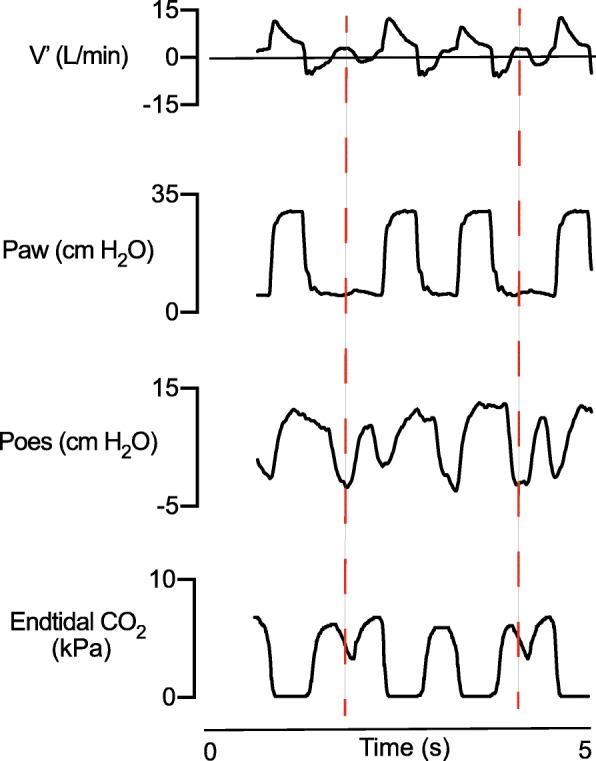
Representative example of ineffective triggering visible in the time-based capnogram. Recording of ventilator flow (V′), airway pressure (Paw), oesophageal pressure (Poes) and end-tidal CO_2_ versus time tracings. The interrupted red line marks an ineffective patient effort. Ineffective triggering visible as a negative deflection in the time-based capnogram can only occur when gas flow that does not contain CO_2_ passes through the sensor. As a consequence, detecting ineffective triggering cannot be done using the time-based capnogram when there is a concomitant flow < 0 L/min. When flow during this effort is becoming > 0 L/min, a negative deflection in the Paw and Poes tracings with a concomitant negative deflection in the time-based capnogram can be seen

**Fig. 2:**
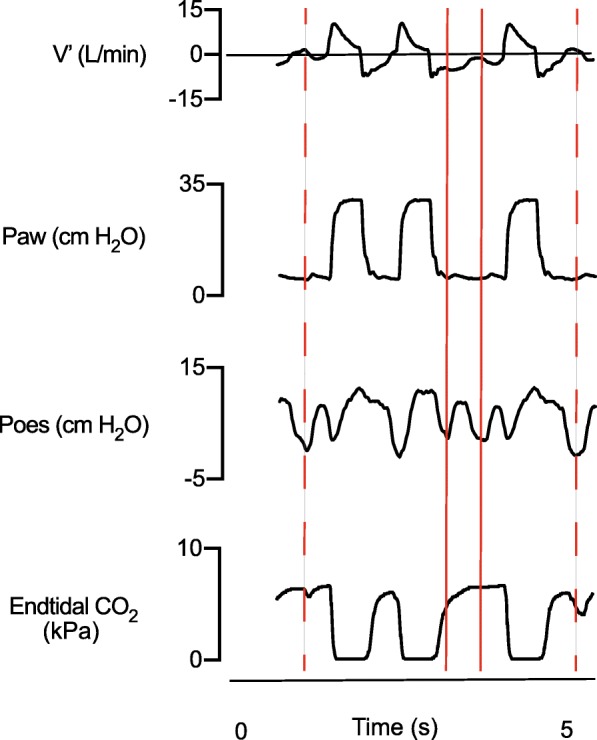
Representative example of ineffective triggering not visible in the time-based capnogram. Recording of ventilator flow (V′), airway pressure (Paw), oesophageal pressure (Poes) and end-tidal CO_2_ versus time tracings. The interrupted red line marks an ineffective patient effort with flow > 0 L/min. The continuous red line marks an ineffective patient effort but the flow remains < 0 L/min. Although a negative deflection is seen in the Paw and Poes tracings, there is no concomitant negative deflection in the time-based capnogram

## Data Availability

The datasets analysed during the current study are available from the corresponding author on reasonable request.

## Abbreviations

dEMG: Transcutaneous measured electrical activity of the diaphragm
Poes: Oesophageal pressure
